# The Biomechanical Response of the Cornea in Orthokeratology

**DOI:** 10.3389/fbioe.2021.743745

**Published:** 2021-10-11

**Authors:** Jinfang Wu, Wenxuan Fang, Huiwen Xu, Xiaode Liu, Dongliang Zhao, Qiguo Rong

**Affiliations:** ^1^ Department of Mechanics and Engineering Science, College of Engineering, Peking University, Beijing, China; ^2^ Department of Epidemiology and Biostatistics, School of Public Health, Peking University, Beijing, China; ^3^ Medical Informatics Center, Peking University, Beijing, China; ^4^ X Lab, the Second Academy of CASIC, Beijing, China; ^5^ School of Chemical Biology and Biotechnology, Peking University Shenzhen Graduate School, Shenzhen, China

**Keywords:** orthokeratology, myopia control, ocular biomechanics, finite element simulation, corneal response

## Abstract

Orthokeratology has been widely used to control myopia, but the mechanism is still unknown. To further investigate the underlying mechanism of corneal reshaping using orthokeratology lenses *via* the finite element method, numerical models with different corneal curvatures, corneal thicknesses, and myopia reduction degrees had been developed and validated to simulate the corneal response and quantify the changes in maximum stress in the central and peripheral corneal areas during orthokeratology. The influence of the factors on corneal response had been analyzed by using median quantile regression. A partial eta squared value in analysis of variance models was established to compare the effect size of these factors. The results showed central and peripheral corneal stress responses changed significantly with increased myopia reduction, corneal curvature, and corneal thickness. The target myopia reduction had the greatest effect on the central corneal stress value (partial eta square = 0.9382), followed by corneal curvature (partial eta square = 0.5650) and corneal thickness (partial eta square = 0.1975). The corneal curvature had the greatest effect on the peripheral corneal stress value (partial eta square = 0.5220), followed by myopia reduction (partial eta square = 0.2375) and corneal thickness (partial eta square = 0.1972). In summary, the biomechanical response of the cornea varies significantly with the change in corneal conditions and lens designs. Therefore, the orthokeratology lens design and the lens fitting process should be taken into consideration in clinical practice, especially for patients with high myopia and steep corneas.

## Introduction

As one of the most effective methods of myopia control, orthokeratology (ortho-k) has been widely used around the world in the past two decades (Wolffsohn et al., 2016; Cho and Tan, 2019; Jonas et al., 2021). The ortho-k lens, a specially designed rigid contact lens, is different from ordinary optical contact lenses because it can change the corneal shape to achieve the purpose of clear vision during the daytime when the lens is removed after overnight wear. The strong effect on myopia control of ortho-k lens for children and adolescents has been proved and reported by numerous clinical researchers. The investigations revealed a slight variance in myopia control effect with an unweighted average 43% (Cho and Cheung, 2012; Hiraoka et al., 2012; Cheung and Cho, 2016; Cho and Cheung, 2017).

Currently, the clinician fits the ortho-k lens based on the patient’s examination results, which include refractive target degree, corneal curvature, corneal e value, and corneal diameter, under the guidance of the manufacturer and clinical experience. Although the effectiveness in reducing myopia and controlling myopia progression of ortho-k lens has been proven by statistical data, the results vary for the individual subjects (Cho et al., 2005). Furthermore, the mechanism by which it controls the development of myopia, the influences, and the weight of these influencing factors are still unknown, while the clinical application of the ortho-k lens has been reported extensively.

Previous researchers had mentioned that the ortho-k lens plays its role mainly *via* the mechanical interaction between the lens and the cornea or the eyeball (Swarbrick, 2004; Wan et al., 2020). Numerous studies (Kobayashi et al., 2008; Lau et al., 2019; Mohidin et al., 2020; Queiros et al., 2020) had attempted to reveal the mechanical effect of wearing ortho-k lenses on the cornea by comparing the change of refractive states, corneal topographies, and corneal thicknesses before and after ortho-k treatment, but few researchers went into details of the analysis due to the complexity of measurement and calculation.

In the current study, finite element modeling was used to analyze the corneal stress responses produced by changing the ortho-k lens parameters and fitting the lenses on various corneas. The purpose of our research was to establish a deeper understanding of the mechanical response of the cornea during ortho-k treatment. The interaction between the lenses and the cornea had been simulated, and the stress responses of the cornea with different designs of ortho-k lens had been analyzed quantitatively. By investigating the corneal stress response, our research strives to reveal the mechanism of ortho-k and contribute to the evaluation of the lens performance and corneal response during the treatment.

## Materials and Methods

### Study Design

The finite element methods were used to analyze the ortho-k process. First, the corneal models were built based on the common corneal thicknesses and corneal curvatures. Second, the corresponding ortho-k lenses were selected in accordance with the manufacturer’s fitting instructions, and the lens parameters were extracted for simulation. Then, the interactions during the wearing process were numerically modeled, and corneal mechanical responses were investigated under these specific conditions. According to the post-fitting changes in the corneal topography, the central area was defined as the central 4 mm area of the corneal apex, outside of which was defined as the peripheral area. The central and peripheral corneal stress under the ortho-k lens was quantified, and the impact of a refractive target, corneal curvature, and corneal thickness on the corneal stress were analyzed.

### Corneal Geometry

Based on the common corneal curvatures and corneal thicknesses, cornea models were developed, with the curvature of 40.25 D, 41.25 D, 42.25 D, 43.25 D, and 44.25 D, respectively, and thicknesses of 500 μm, 525 μm, 550 μm, and 575 μm, respectively, as shown in Table 1. The corneal radius was calculated by the following formula:

$$r=\frac{1000\times \left(n-1\right)}{D},$$ (1)

where n is the refractive index of the cornea ( n =1.337), D is the corneal refractive power, and r is the corneal curvature radius. A total of 20 ideal cornea models were established for the finite element analysis.

**TABLE 1:** Corneal curvature, corneal thickness, and myopia.

Variable	Value				
Corneal curvature (D)	40.25	41.25	42.25	43.25	44.25
Corneal thickness (μm)	500	525	550	575	
Myopia (D)^a^	−2.00	−3.00	−4.00	−5.00	

a The degree of myopia used for lens selection.

### Ortho-k Lens Geometry

**FIGURE 1:**
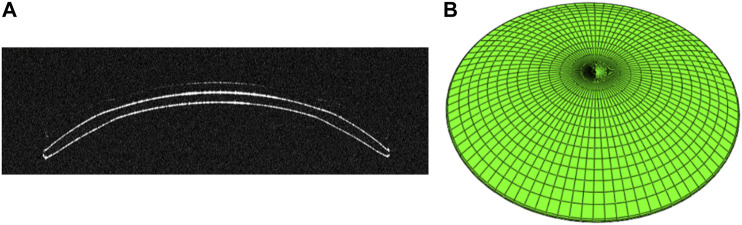
**(A)** OCT B-Scan image of ortho-k lens from the Optimec is830. **(B)** Three-dimensional model of ortho-k lens.

Four-zone ortho-k lenses (α ortho-k; Alpha Corp., Nagoya, Japan) made of highly oxygen permeable lens material (Boston EM, nominal Dk of 104 × 10^–11^ (cm^2^/sec) (mL O_2_/mL × mmHg)) were used in this study. Based on the parameters of the corneal curvatures (40.25 D, 41.25 D, 42.25 D, 43.25 D, and 44.25 D) and the target myopia reduction degrees (−2.00 D, −3.00 D, −4.00 D, and −5.00 D), twenty commonly used ortho-k lens parameters were selected for the investigation, as shown in Table 2. The two-dimensional geometry of the ortho-k lenses was measured by a noncontact optical coherence tomography (OCT) metrology system, the Optimec *is*830 (Optimec Systems Limited, United Kingdom), in four meridians (0°, 90°, 180°, and 270°) (Coldrick et al., 2016). The lens’ thickness, diameter, sagittal data, and all of the curvatures of each zone were extracted. The geometry model is shown in Figure1A. The average coordinates of the four meridians captured by OCT were used to rotate into three-dimensional models of the ortho-k lenses using SolidWorks software (SOLIDWORKS 2017, Dassault Systemes, the United States), as shown in Figure 1B.

**TABLE 2:** The selected lens parameters.

Item	Flat K (D)	Diameter (mm)	BCR (mm)	BCRP (D)	CF (D)	CT (mm)	Myopia (D)
1	40.25	10.6	9.00	37.50	+0.75	0.22	−2.00
2	40.25	10.6	9.25	36.50	+0.75	0.22	−3.00
3	40.25	10.6	9.51	35.50	+0.75	0.22	−4.00
4	40.25	10.6	9.78	34.50	+0.75	0.22	−5.00
5	41.25	10.6	8.77	38.50	+0.75	0.22	−2.00
6	41.25	10.6	9.00	37.50	+0.75	0.22	−3.00
7	41.25	10.6	9.25	36.50	+0.75	0.22	−4.00
8	41.25	10.6	9.51	35.50	+0.75	0.22	−5.00
9	42.45	10.6	8.50	39.70	+0.75	0.22	−2.00
10	42.45	10.6	8.72	38.70	+0.75	0.22	−3.00
11	42.45	10.6	8.95	37.70	+0.75	0.22	−4.00
12	42.45	10.6	9.20	36.70	+0.75	0.22	−5.00
13	43.25	10.6	8.33	40.50	+0.75	0.22	−2.00
14	43.25	10.6	8.54	39.50	+0.75	0.22	−3.00
15	43.25	10.6	8.77	38.50	+0.75	0.22	−4.00
16	43.25	10.6	9.00	37.50	+0.75	0.22	−5.00
17	44.25	10.6	8.13	41.50	+0.75	0.22	−2.00
18	44.25	10.6	8.33	40.50	+0.75	0.22	−3.00
19	44.25	10.6	8.54	39.50	+0.75	0.22	−4.00
20	44.25	10.6	8.77	38.50	+0.75	0.22	−5.00

Flat K: flat keratometry; D: degree; BCR: base curve radius; BC: base curve refractive power; CF: compression factor; CT: central thickness.

### Finite Element Model

**FIGURE 2:**
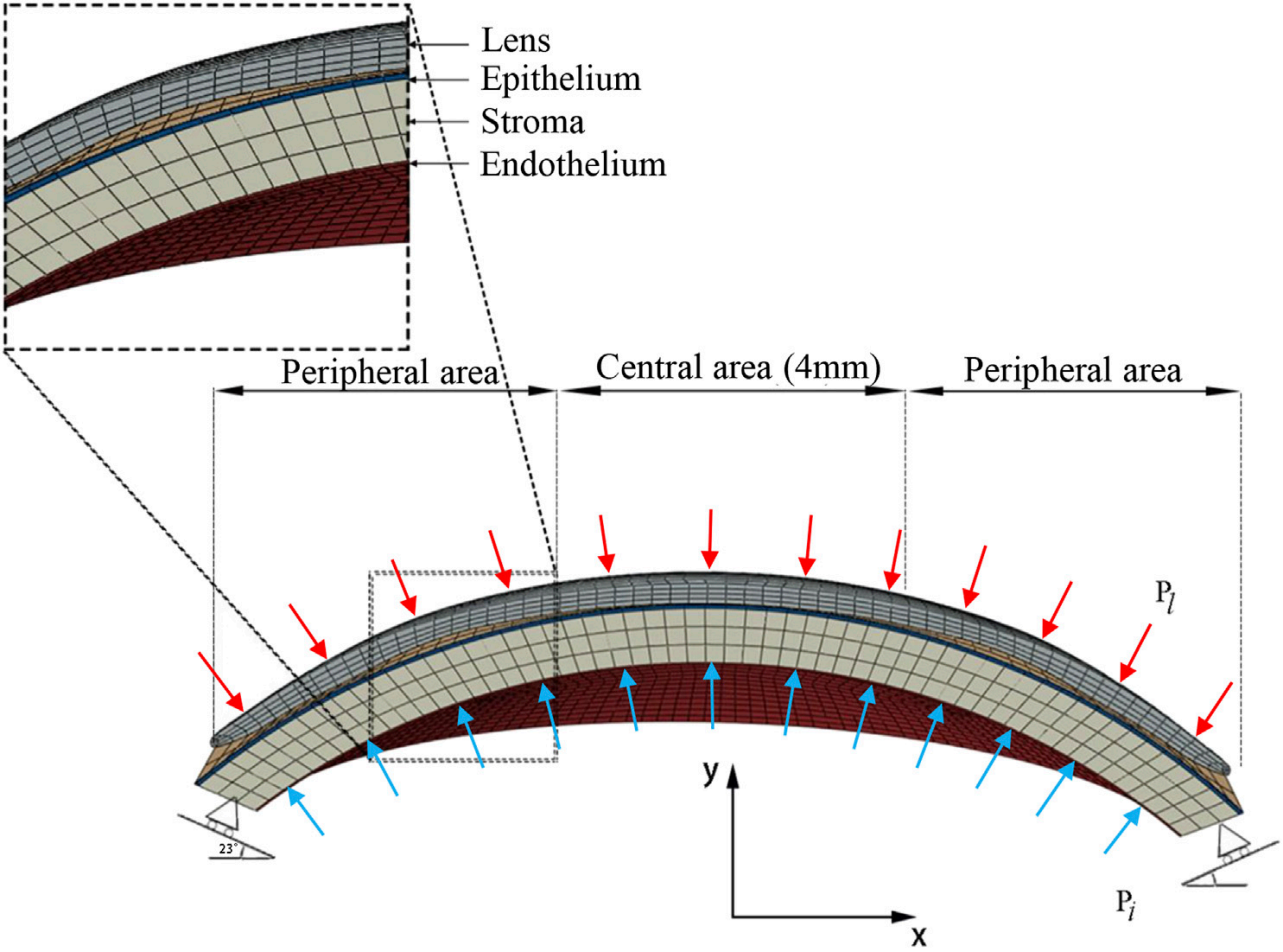
FEA models of ortho-k lens and the cornea.

Histologically, the microanatomical structure of the cornea can be divided into five layers from the anterior to the posterior: epithelium, Bowman’s membrane, stroma, Descemet’s membrane, and endothelium. In our modeling of the cornea, three layers of different elastic modulus were assigned to represent the real corneal structure. The corneal epithelium and Bowman’s membrane were regarded as one layer, as was Descemet’s membrane and endothelium. The layer thickness of the epithelial and Bowman’s membrane was presumed to be 50–57.5 μm, 10 percent of the total corneal thickness, as in routine clinical findings (Ehlers et al., 2010). The cornea was assumed to be a nonhomogeneous elastic material (Pinsky et al., 2005; Elsheikh et al., 2009; Last et al., 2009), and all material properties are shown in Table 3 (Wollensak et al., 2003; Pinsky et al., 2005; Elsheikh et al., 2008; Elsheikh et al., 2009; Last et al., 2009). According to the previous studies (Elsheikh et al., 2008; Elsheikh et al., 2009), the stiffness of both the outermost and innermost layers were assumed to be 10 percent as stiff as the corneal stroma. The finite element mesh of structured elements was generated. A mesh dependency study was conducted such that the relative error in two consecutive mesh refinements was <0.5% for computation. The mesh and boundary conditions are shown in Figure 2. Clinical studies have proven that principally only the thickness of the corneal epithelium and the curvature of the anterior cornea are changed when wearing an ortho-k lens, without bending of the entire cornea or affecting the depth of the anterior chamber (Alharbi and Swarbrick, 2003; Kim et al., 2018; Swarbrick et al., 2020). Therefore, in our model, the sclera was constrained from moving in all directions. To best match with the model of the whole eye, the orientation of corneal edge support was set to 23 (Elsheikh and Wang, 2007). The intraocular pressure (IOP) was set to 13 mmHg and homogeneously applied on the inner corneal surface, while the eyelid pressure was set to 9 mmHg and applied on the outer surface of the lens (Shaw et al., 2010). Eighty static finite element simulations were performed using the Abaqus software (version 6.14 Dassault Systemes, Simulia Corp., RI, the United States) for revealing the stress and displacement status of ortho-k lenses’ wearing.

**TABLE 3:** Lens and cornea material properties.

	Young’s modules	Poisson ratio
Ortho-k lenses	100 MPa	0.3
Outermost layer*	127 kPa	0.49
Interlayer*	1270 kPa	0.49
Innermost layer*	127 kPa	0.49

Outermost layer: epithelium and Bowman’s membrane; interlayer: corneal stroma; innermost layer: Descemet’s membrane and endothelium.

### Statistical Analysis

The normality of residuals and homogeneity of variance were tested by using the Shapiro–Wilk test and Levene’s test, respectively. Because data were found to violate normality, median quantile regression was conducted to explore factors influencing central corneal stress and peripheral corneal stress. Quantile regression is an approach that models the distribution of the responses and performs better than conventional means of regression when the normality assumption is violated. The central corneal stress value and the peripheral corneal stress value were, respectively, used as dependent variables (continuous variables), and the degree of myopia, corneal curvature, and corneal thickness were included as independent variables (categorical variables) in the models. Furthermore, to estimate the effect size of the independent variables on dependent variables, we used the analysis of variance (ANOVA) method and reported partial eta square as the measure of effect size. Partial eta squared ( $partial\;\eta^{2})$ describes the ratio of variance explained in the dependent variable by an independent variable while controlling other independent variables. It is defined as

$$partial\;\eta^{2}=\frac{SS_{effect}}{SS_{effect}+SS_{error}}.$$ (2)

Since residuals in the stress value model are non-normally distributed and residuals in the curvature change do not have constant variance, the Box–Cox transformation was performed on the stress value and the curvature change to meet the ANOVA assumptions. The Box–Cox transformation can obtain a normal distribution of the transformed data (after transformation) and a constant variance. The one-parameter Box–Cox transformation is defined as

$$y_{i}^{\left(\lambda \right)}=\{\begin{array}{c} \frac{\;\;y_{i}^{\lambda }-1\;\;}{\lambda }\;\;\;\;\;\;\;\;\;\;if\;\lambda \;\neq 0\\ \ln y_{i}\;\;\;\;\;\;\;\;\;\;\;\;\;\;\;\;\;\;if\;\lambda \;=0 \end{array},$$ (3)

and the two-parameter Box–Cox transformations as

$$y_{i}^{\left(\lambda \right)}=\{\begin{array}{c} \frac{\;\;{\left(y_{i}+\lambda_{2}\right)}^{\lambda_{1}}-1\;\;}{\lambda_{1}}\;\;\;\;\;\;\;\;\;\;if\;\lambda_{1}\neq 0\\ \ln\left(y_{i}+\lambda_{2}\right)\;\;\;\;\;\;\;\;\;\;\;\;\;\;\;\;\;\;\;\;\;\;if\;\lambda_{1}=0 \end{array}\;.$$ (4)

Moreover, the first transformation holds for $y_{i}>0$ and the second for $y_{i}>-\lambda_{2}$ . All statistical analyses were performed by SAS 9.4 (SAS Institute, Inc., Cary, NC), and two-sided *p*-values of less than 0.05 were considered statistically significant.

## Results

### Corneal Biomechanical Responses Under ortho-k With FEA

**FIGURE 3:**
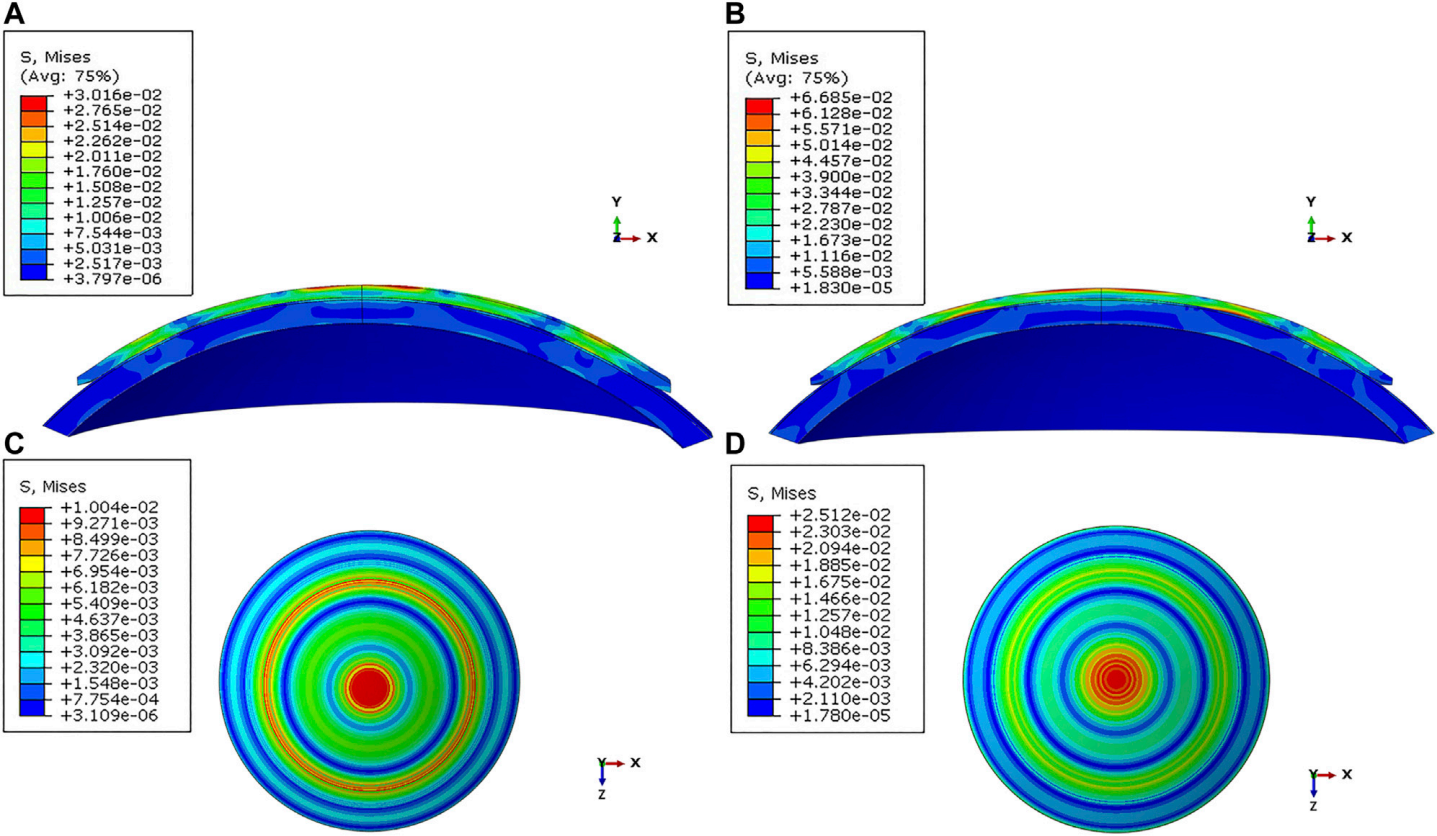
Distribution of the von Mises stress in the cornea under varied conditions. **(A)** Sagittal face: −2.00 D/40.25 D/500 μm; **(B)** Sagittal face: −5.00 D/44.25 D/575 μm; **(C)** En face: −2.00 D/40.25 D/500 μm; **(D)** En face: −5.00 D/44.25 D/575 μm.

In the current study, the central corneal stress and the peripheral corneal stress were regarded as continuous variables, while myopia, corneal curvature, and corneal thickness were regarded as categorical variables. The maximum von Mises stress in the central corneal area and the peripheral area are shown in Table 4 and Table 5. It can be found that the maximum von Mises stress of the corneal central area showed an upward trend with the increase of myopia reduction degree, corneal curvature, and corneal thickness. The average of central cornea stress was 0.019178 MPa (SD = 0.017438). For the central cornea, wearing a −2.00 D ortho-k lens with a curvature of 40.25 D and a corneal thickness of 500 μm had the smallest maximum von Mises stress (0.01035 MPa), while wearing a −5.00 D ortho-k lens with a curvature of 44.25 D and a thickness of 575 μm had the largest maximum von Mises stress (0.02513 MPa). It can be seen that the maximum von Mises stress of different ortho-k lenses with different degrees increased by 2.42 times in the corneal center. Figure 3 shows the distribution of the maximum von Mises stress in the cornea with different corneal thickness, curvature, and refractive change.

**TABLE 4:** Maximum stress in the central corneal area (MPa).

Myopia degree: 2.00 D				
**Corneal curvature (D)/thickness (μm)**	**500**	**525**	**550**	**575**
**40.25**	0.01035	0.01039	0.01050	0.01089
**41.25**	0.01107	0.01132	0.01154	0.01162
**42.25**	0.01128	0.01151	0.01183	0.01219
**43.25**	0.01167	0.01186	0.01200	0.01219
**44.25**	0.01238	0.01256	0.01270	0.01289
**Myopia degree: 3.00 D**				
**Corneal curvature (D)/thickness (μm)**	**500**	**525**	**550**	**575**
**40.25**	0.01356	0.01379	0.01405	0.01482
**41.25**	0.01407	0.01432	0.01456	0.01491
**42.25**	0.01480	0.01510	0.01562	0.01597
**43.25**	0.01527	0.01586	0.01605	0.01619
**44.25**	0.01608	0.01636	0.01662	0.16810
**Myopia degree: −4.00 D**				
**Corneal curvature (D)/thickness (μm)**	**500**	**525**	**550**	**575**
**40.25**	0.01663	0.01682	0.01695	0.01707
**41.25**	0.01719	0.01826	0.01852	0.01881
**42.25**	0.01853	0.01887	0.01917	0.01951
**43.25**	0.02036	0.02094	0.02151	0.02206
**44.25**	0.02166	0.02194	0.02216	0.02237
**Myopia degree: −5.00 D**				
**Corneal curvature (D)/thickness (μm)**	**500**	**525**	**550**	**575**
**40.25**	0.02021	0.02057	0.02146	0.02258
**41.25**	0.02127	0.02196	0.02260	0.02291
**42.25**	0.02190	0.02233	0.02297	0.02351
**43.25**	0.02286	0.02325	0.02354	0.02373
**44.25**	0.02402	0.02448	0.02477	0.02513

**TABLE 5:** Maximum stress in the peripheral corneal area (MPa).

Myopia degree: −2.00 D							
**Corneal curvature (D)/thickness (μm)**	**500**	**525**	**550**	**575**
**40.25**	0.01146	0.01093	0.01058	0.00985
**41.25**	0.01234	0.01208	0.01183	0.01063
**42.25**	0.01345	0.01247	0.01270	0.01158
**43.25**	0.01374	0.01331	0.01299	0.01250
**44.25**	0.01733	0.01682	0.01610	0.01552
**Myopia degree: −3.00 D**				
**Corneal curvature (D)/thickness (μm)**	**500**	**525**	**550**	**575**
**40.25**	0.01236	0.01187	0.01163	0.01121
**41.25**	0.01317	0.01266	0.01228	0.01190
**42.25**	0.01381	0.01321	0.01267	0.01238
**43.25**	0.01472	0.01401	0.01380	0.01335
**44.25**	0.01706	0.01674	0.01631	0.01582
**Myopia degree: −4.00 D**				
**Corneal curvature (D)/thickness (μm)**	**500**	**525**	**550**	**575**
**40.25**	0.01335	0.01308	0.01261	0.01187
**41.25**	0.01301	0.01267	0.01184	0.01119
**42.25**	0.01392	0.01336	0.01291	0.01260
**43.25**	0.01519	0.01444	0.01382	0.01358
**44.25**	0.01699	0.01513	0.01468	0.01425
**Myopia degree: −5.00 D**				
**Corneal curvature (D)/thickness (μm)**	**500**	**525**	**550**	**575**
**40.25**	0.01273	0.01227	0.01158	0.01175
**41.25**	0.01304	0.01245	0.01176	0.01189
**42.25**	0.01309	0.01269	0.01226	0.01190
**43.25**	0.01324	0.01273	0.01234	0.01215
**44.25**	0.01358	0.01289	0.01274	0.01253

**FIGURE 4:**
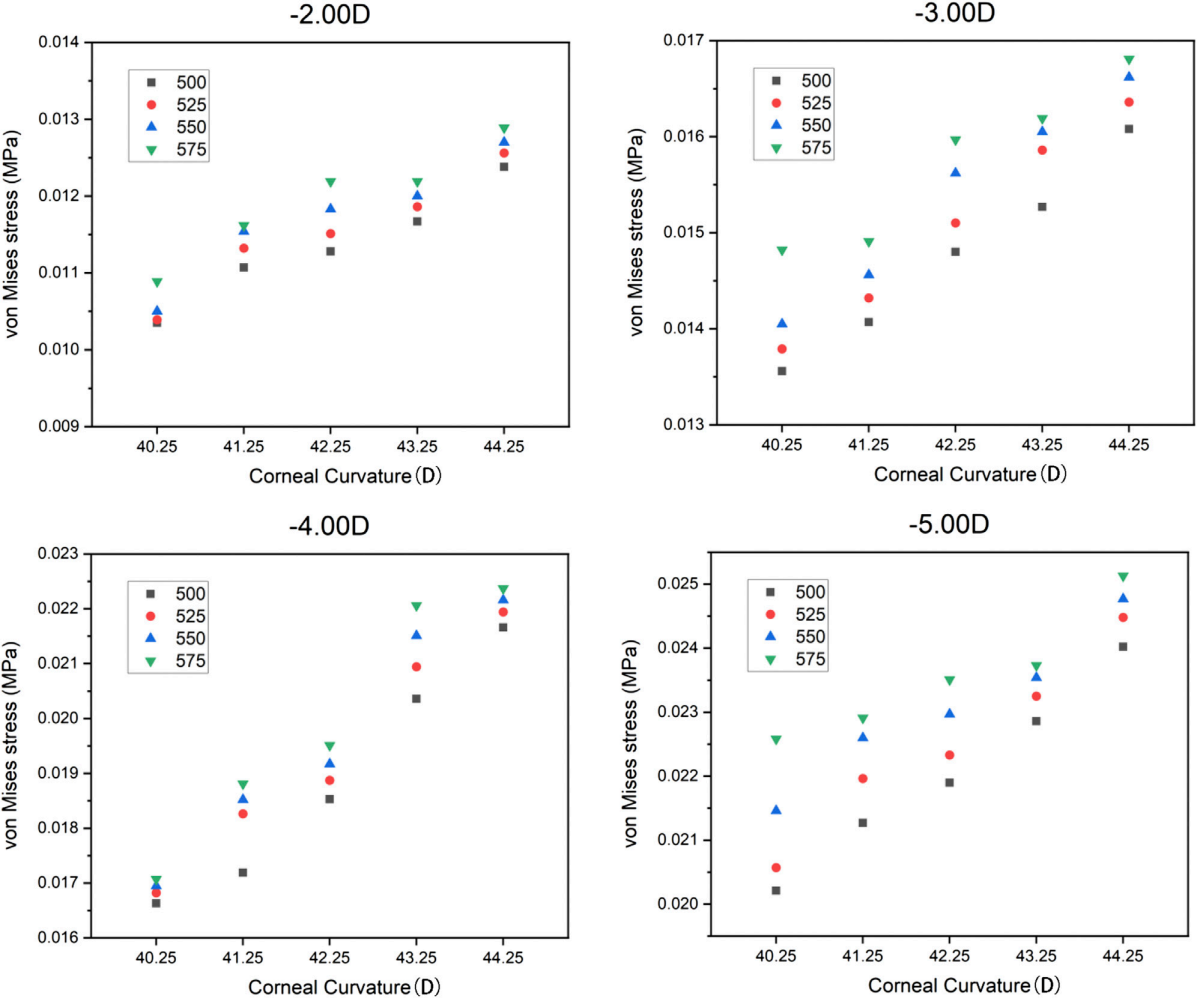
Representative maximum von Mises stress in the central corneal area.

**FIGURE 5:**
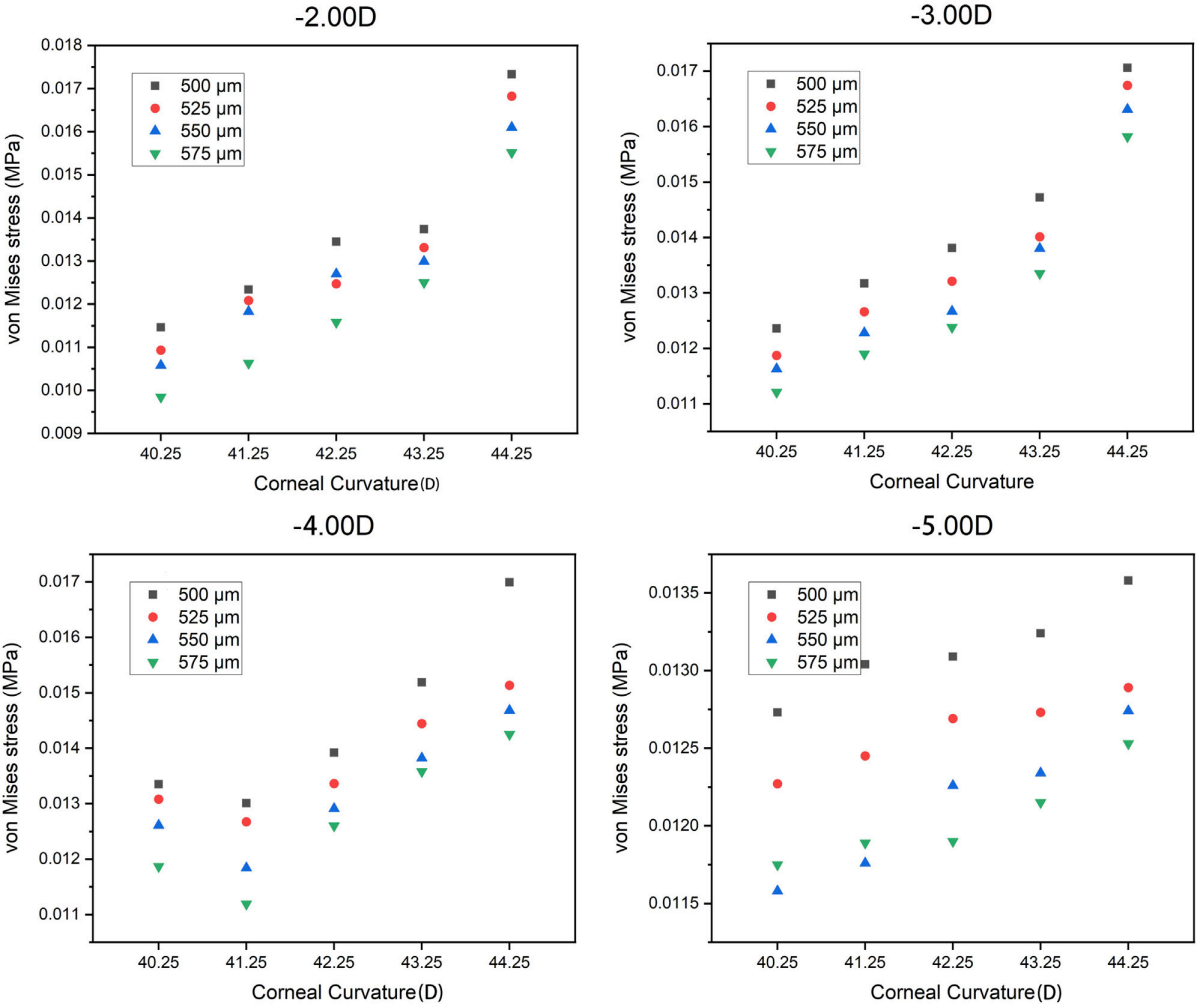
Representative maximum von Mises stress in the peripheral corneal area.

The trend of the maximum von Mises stress in the peripheral cornea was opposite to that in the corneal center. As the cornea thickness increased, the maximum von Mises stress in the peripheral area decreased gradually under the same curvature. The change of the maximum von Mises stress under various degrees was not large, and the maximum von Mises stress ranged from 0.00985 to 0.01733 MPa, with an average stress of the 80 models being 0.014451 MPa (SD = 0.011908). For −3.00 D, −4.00 D, and −5.00 D lens wearing, the maximum von Mises stress occurred in the central cornea, while for −2.00 D, the maximum von Mises stress was at the peripheral cornea. The representative maximum von Mises stress appearing in the central and peripheral areas are shown in Figure 4 and Figure 5.

### Quantile Regression Results of Dependent Variables and Independent Variables

The quantile regression results of central corneal stress and independent variables are shown in Table 6. Compared with myopia of −2.00 D, the central corneal stress value increased when the myopia was −3.00 D (*β* = 0.0036, *p* < 0.001), −4.00 D (*β* = 0.0074, *p* < 0.01), and −5.00 D (*β* = 0.0111, *p* < 0.01). Compared with corneal curvature of 40.25 D, the central corneal stress value increased when the corneal curvature was 41.25 D (*β* = 0.0007, *p* < 0.01), 42.25 D (*β* = 0.0014, *p* < 0.01), 43.25 D (*β* = 0.0019, *p* < 0.01), and 44.25 D (*β* = 0.0031, *p* < 0.01). Compared with a corneal thickness of 500 μm, the central corneal stress value increased when the corneal thickness was 525 μm (*β* = 0.0004, *p* = 0.0298), 550 μm (*β* = 0.0007, *p* < 0.01), and 575 μm (*β* = 0.0011, *p* < 0.01). All models were controlled for the other covariates.

**TABLE 6:** Quantile regression results of central corneal stress and independent variables.

Variables	Category	β	*p*-value
**Myopia (D)**	−2.00	Ref	Ref
	−3.00	0.0036	<0.001
	−4.00	0.0074	<0.001
	−5.00	0.0111	<0.001
**Corneal curvature (D)**	40.25	Ref	Ref
	41.25	0.0007	0.0072
	42.25	0.0014	<0.001
	43.25	0.0019	<0.001
	44.25	0.0031	<0.001
**Corneal thickness (μm)**	500	Ref	Ref
	525	0.0004	0.0298
	550	0.0007	<0.001
	575	0.0011	<0.001

The quantile regression results of corneal peripheral stress and independent variables are shown in Table 7. In patients with myopia of −3.00 D (*β* = 0.0007, *p* < 0.01) and −4.00 D (*β* = 0.0009, *p* < 0.01), the corneal peripheral stress increased compared with patients with myopia of −2.00 D. In patients with corneal curvature of 42.25 D (*β* = 0.0007, *p* = 0.0324), 43.25 D (*β* = 0.0015, *p* < 0.01), and 44.25 D (*β* = 0.0040, *p* < 0.01), the peripheral corneal stress value increased compared with patients with corneal curvature of 40.25 D. In patients with corneal thickness of 525 μm (*β* = −0.0005, *p* < 0.01), 550 μm (*β* = −0.0009, *p* < 0.01), and 575 μm (*β* = −0.0013, *p* < 0.01), the peripheral corneal stress value decreased compared with patients with corneal thickness of 500 μm. All models were controlled for the other covariates.

**TABLE 7:** Quantile regression results of corneal peripheral stress and independent variables.

Variables	Category	β	*p*-value
**Myopia (D)**	−2.00	Ref	Ref
	−3.00	0.0007	<0.001
	−4.00	0.0009	0.0001
	−5.00	0.0002	0.5713
**Corneal curvature (D)**	40.25	Ref	Ref
	41.25	0.0001	0.7053
	42.25	0.0007	0.0324
	43.25	0.0015	<0.001
	44.25	0.0040	<0.001
**Corneal thickness (μm)**	500	Ref	Ref
	525	−0.0005	0.0037
	550	−0.0009	<0.001
	575	−0.0013	<0.001

### Comparison of the Effect of Each Independent Variable on the Dependent Variable

The larger the partial eta value, the greater the effect of the independent variable on the dependent variable. The results of the variance (ANOVA) method are as follows: The degree of myopia had the greatest effect on the central corneal stress value (partial eta square = 0.9382), followed by corneal curvature (partial eta square = 0.5650) and corneal thickness (partial eta square =  0.1975). The curvature of the cornea had the greatest effect on the peripheral stress value of the cornea (partial eta square =  0.5220), followed by myopia (partial eta square =  0.2375) and corneal thickness (partial eta square =  0.1972).

## Discussion

The rapidly growing myopic prevalence has sparked intense research interest in the methods of myopia control (Cho and Cheung, 2012; Gonzalez-Meijome et al., 2016; Bremond-Gignac, 2020; Bullimore and Johnson, 2020). Ortho-k, a most promising treatment approach to slow down myopia progression, is gaining popularity among myopic patients due to its effectiveness and convenience. By using specially designed gas-permeable contact lenses to reshape the cornea, ortho-k has an effect in temporarily correcting myopia and controlling myopia progression (Cho et al., 2008; Hiraoka et al., 2012). However, the corneal integrity can be compromised if an improperly designed lens is fitted (Li S.-M. et al., 2016; Cheng et al., 2016; Bullimore et al., 2021). Therefore, there is an urgent need to understand the biomechanical change during ortho-k treatment and to identify the population most suitable for this treatment.

Currently all the existing theoretical hypotheses that the ortho-k lens controls myopia progression, such as the retinal peripheral defocus theory (Gardner et al., 2015; Smith et al., 2020), the aberration theory (Hiraoka et al., 2015; Zhang X. et al., 2020; Lau et al., 2020), and the accommodation theory (Batres et al., 2020; Song et al., 2021), are based on the change of the corneal shape or reduction of the refractive error after ortho-k lens wear. The biomechanical interaction between the lens and the cornea or the entire eyeball is vitally important in the course of ortho-k treatment. Different from the regular rigid contact lenses, the ortho-k lens has a special design, which is reversed against the corneal surface to reshape the cornea. The difference in the lens parameters will create different pressure to the cornea. Under the action of this force, the corneal morphology changes. Then, the lens can achieve its effects of reducing refractive error and controlling the myopia progression. However, the force of the interaction between the lens and cornea, or the pressure under the ortho-k lens on the cornea is difficult to measure or calculate. To the best of the authors’ knowledge, the research in this field remains very limited. Therefore, mathematical modeling quantifying the pressure and force that the cornea receives can play a key role to reveal the mechanism.

A finite element analysis (FEA), a commonly used numerical method, is an effective way to study and analyze the mechanical behavior and functional mechanism of the cornea, which has been extensively implemented to study corneal mechanical problems (Kling et al., 2014; Li ZD. et al., 2016; Fang et al., 2020; Meng et al., 2020). Not only has the FEA been widely recognized for its potential in simulating and analyzing the effects of corneal surgery, it has also been used in corneal diseases and ocular trauma because of its predictive feature. The focus of our current work was to use the FEA to analyze the biomechanical response of the cornea under the ortho-k lens. Based on the finite element models, this article compared and analyzed the von Mises stress in different areas of the corneas of various thicknesses and curvatures within the wearing course of a variety of ortho-k lens designs.

The whole-eye model with both the cornea and the sclera would provide a better representation of the actual state. However, it was extremely time-consuming. Some studies showed that if the boundary conditions were defined properly along the corneal edge, the cornea-only models can represent the whole-eye model very well. We set the orientation of corneal edge support to 23 in FEA according to Elsheikh and Wang (2007). The final results of our study were fair and showed consistent trends with clinical studies. In our model, the selection ranges of corneal curvature and corneal thickness were based on common clinical values. Although dividing the cornea into five layers according to the physiological structure can better show its mechanical response, due to the lack of relevant research data, it is impossible to accurately define the material properties of each layer and the interaction between the layers. Many articles showed that the corneal changes after wearing the ortho-k lens were mainly confined to the epithelial layer (Kim et al., 2018; Mohidin et al., 2020). Furthermore, the other layers of the cornea are relatively much thinner than the stromal layer, which may cause little effect on the simulation results. Therefore, the hypothesis of the three-layer corneal model is reasonable. Recently, many studies performed the corneal simulation using the hyper-elastic material model (Elsheikh and Wang, 2007; Bao et al., 2018). However, the behavior of a nonlinear material model is very similar to that of the elastic material model under 13 mmHg (1.73 KPa) IOP (Elsheikh and Wang, 2007). Nonlinear elastic materials can deform linearly for very small deformation (Wollensak et al., 2003). In order to improve the simulation’s efficiency, the corneal material was assumed to be elastic in this study.

According to the above results, the change of the targeted myopia reduction degree had the greatest impact on corneal stress. In the ortho-k lens design, the reduction degree mainly affects the base curve radius (BCR), a means of controlling the refractive change, which makes the cornea flatter if the target reduction is increased and base curve becomes flatter. From a biomechanical point of view, increasing the target degree means applying more compressive pressure on the corneal center. We analyzed the corneal responses with the change of targeted reduction degree. As the refractive target increased, both the central and peripheral corneal stress responses increased significantly, especially in the corneal center. Although the FDA or other health authorities approve the use of ortho-k treatment in myopia up to −6.00 D in some lens designs, the safest and most successful outcome will be achieved for those patients with lower baseline levels of myopia (Mika et al., 2007; Liu and Xie, 2016; Singh et al., 2020; Hu et al., 2021). Therefore, for patients with high myopia or for doctors who used to increase the target degrees, more attention must be paid to the possible side effects of the ortho-k lens wear, such as damage to the integrity of the cornea, since those lenses are designed with increased compressive pressure.

The corneal curvature is a very important parameter in ortho-k lens design. The calculation of BCR and the design of the lens alignment curve are based on the corneal curvature. The range of the corneal curvatures for most patients is between 40 and 46 D, and the most common is between 42 and 43 D. If the cornea is too flat or too steep, it can cause problems during lens fitting. When the cornea is too flat, the range of corneal deformation is limited, and adequate treatment may not be achieved, especially for high myopia. However, if the corneal is too steep, the incident of corneal staining is higher than that of the common corneal curvature (Cheng et al., 2016). In our study, it was found that the cornea had a larger stress response when the corneal curvature became steeper for both the central and peripheral areas. Therefore, practitioners need to be more cautious in treating patients with steep corneas and high myopia for safety concerns.

From our research results, it can be seen that corneal thickness also affects corneal stress in the central and peripheral areas. However, in the normal range, the corneal thickness is the factor that has the least influence on the corneal stress response among all the factors. After wearing ortho-k lenses, the corneal thickness has a certain amount of thinning (Wan et al., 2021). However, a large number of studies have found that the changes of corneal thickness are mainly confined to the corneal epithelium (Kim et al., 2018; Zhang J. et al., 2020; Zhou et al., 2020a; b). Therefore, it is relatively safe to wear an ortho-k lens within the range of normal corneal thickness.

There are some limitations in the current study. First, our model was mainly designed to analyze the positive pressure of the lens on the cornea without analyzing the tear layer force. Factors such as squeeze film force, or tension force, which are related to the tear viscosity and the tear thickness under the lens, are important in helping to alter the corneal shape and ideally centralize the lens position. In the future research, we will establish the tear flow field and analyze the tear layer under the lens. Second, this research assumed the cornea to be an ideal sphere. However, there have been some controversy about the changes of corneal shape during ortho-k, and the initial corneal eccentricity may affect lens selection. Creating the definite model of the eyeball according to the actual shape of the human cornea could be the foundation for the individual predictive model. Third, in this study, we were trying to reveal the biomechanical response of the cornea in the ortho-k treatment under the condition of various corneal shapes and thicknesses, and a refractive change with the uniform setting criteria of the pressures, including the IOP and eyelid tension. However, the lid tension and IOP may have potential effects on ortho-k. Therefore, we will explore the influence of these factors on the corneal mechanical response during ortho-k treatment in the future.

## Conclusion

This article investigated the interaction between the cornea and the ortho-k lens through finite element methods to clearly reveal the mechanical response of the cornea under ortho-k. The current work provides ortho-k lens practitioners with an objective, reliable, and noncontact approach for quantitative assessment of risk and effectiveness of ortho-k for specific patients. This research also provides insights into the design of future ortho-k lenses. In ortho-k treatment, both the refractive target and the corneal curvature have a significant impact on corneal stress. Any change in corneal thickness within the normal range has minimal impact on stress. In clinical practice, more attention should be paid to patients with high myopia and high corneal curvature.

## Data Availability

The original contributions presented in the study are included in the article/Supplementary Material; further inquiries can be directed to the corresponding authors.
